# Use of a Chemotherapy Toxicity Prediction Tool to Decrease Risks for Hospitalization in Older Patients

**DOI:** 10.7759/cureus.24465

**Published:** 2022-04-25

**Authors:** Mintcho E Mintchev, Arjun G Kalra, Chung-Ting J Kou, James K Aden, Matthew L Bezzant, Wilfred P dela Cruz, Adrian R Bersabe

**Affiliations:** 1 Hematology and Oncology Service, Brooke Army Medical Center, Fort Sam Houston, USA; 2 Internal Medicine Service, Brooke Army Medical Center, Fort Sam Houston, USA; 3 Statistics, Brooke Army Medical Center, Fort Sam Houston, USA; 4 Hematology/Oncology, Keesler Air Force Base Hospital, Biloxi, USA

**Keywords:** risks for hospitalization, eastern cooperative oncology group (ecog), quality improvement research, geriatric oncology, medical frailty, older adult, chemotherapy-related toxicity, military medicine, treatment decisions, geriatric assessment

## Abstract

Objectives

Performance status (PS) scales such as the Eastern Cooperative Oncology Group (ECOG) PS and the Karnofsky Performance Index have limited utility in selecting therapies and predicting related adverse events in older patients with cancer. In July 2016, medical oncologists at our institution adopted the Cancer and Aging Research Group toxicity prediction score (CARG), a toxicity prediction tool, to identify patients who are “fit” for chemotherapy versus those who are “frail” and may experience severe complications.

Methods

Our retrospective review included referrals of beneficiaries 75 years of age and older who received standard systemic therapy and patients of the same age whose treatment was modified due to CARG. We compared the score’s utilization six months before and after its incorporation and then assessed how its application impacted admissions, emergency department (ED) visits, and medical management.

Results

Thirty-eight patients with a mean age of 81 years met the inclusion criteria. Their diagnoses included gastrointestinal (37%), lung (21%), hematologic (18%), breast (10.5%), genitourinary (3%), and other (10.5%) malignancies. CARG was documented for 12.5% of systemic therapy recipients before its adoption and 41% of recipients after adoption. Its use was limited by the reliance on physicians to perform scoring during time-constrained patient encounters. Patients had fewer mean inpatient admissions (0.7 versus 2.3), admission days (4.3 versus 8), and ED visits (1.1 versus 2.5) when management was modified based on the score.

Conclusion

CARG assessment may facilitate a safer and more tailored approach to cancer care in older patients than conventional PS scales alone. Its integration into patient screening would increase its application and better define its potential predictive capacity to decrease risks for hospitalization.

## Introduction

Aging is the single greatest risk factor for cancer, with an 11-fold increase in the incidence of cancer in persons 65 years of age and older compared with younger people [1,2]. It is estimated that older patients will comprise 20% of the United States population by 2030, and this rapidly growing population has a 16-fold increase in cancer mortality compared with younger people [1,2]. However, patients with cancer who are 75 years of age and older are markedly underrepresented in clinical trials [3-5]. The utility of performance status (PS) scales such as the Eastern Cooperative Oncology Group (ECOG) PS and the Karnofsky Performance Index in the oncologic care of this population is not well studied. These conventional measures have been largely validated in younger patients to help determine eligibility for cancer treatments, but they do not take into account the physiologic effects of the aging process and common comorbidities that may increase vulnerability to therapy-related toxicity [2]. They also have limited predictive value in selecting appropriate systemic therapy regimens and considering therapy-related morbidity and mortality [6]. Moreover, physicians’ instincts and patients’ determination for treatment could make conventional PS assessments prone to bias and substantially degrade the objective assessment of frailty.

There is a general acceptance among oncologists that older patients with cancer belong to a heterogeneous population with unique circumstances that may affect their response and tolerance to cancer treatments. Consequently, it is difficult to develop a standard approach when evaluating these individuals [2,6]. The National Comprehensive Care Network® suggests the use of several tools to facilitate appropriate evaluations of older patients with cancer who may be able to tolerate treatment. One of these is the Cancer and Aging Research Group toxicity prediction score (CARG), which may be conveniently determined using an online medical calculator. Its model was studied in a large, prospective, multicenter study and has subsequently been validated in predicting the probability of grade 3-5 chemotherapy-related toxicity in older adults [2]. Age, cancer type, hemoglobin, creatinine, and several geriatric assessment variables were identified as risk factors for chemotherapy-related toxicity. Additionally, the model enables clinicians to compare the probabilities for toxicities of various chemotherapy regimens through its incorporation of dosage and number of chemotherapy agents as risk factor variables.

CARG is a practical tool that can help spare older patients from potentially lethal complications from therapy or reach acceptable levels of risk through a well-informed reduction in treatment intensity. There is limited data on how the use of CARG influences management and outcomes; therefore, the extent of its role in geriatric oncology remains uncertain. Moth et al. helped describe this uncertainty by showing that 83% of medical oncologists found CARG useful when it was provided to them, but they rarely used it to modify chemotherapy [7]. We performed this study to assess our quality improvement efforts aimed at introducing CARG to our institution and to aid us in standardizing its use in caring for older patients. Our primary goal was to evaluate the frequency of its utilization and its ease of adoption, but we also assessed whether basing patient management decisions on it decreased risks for hospitalization in terms of admissions, admission days, and emergency department (ED) visits.

## Materials and methods

Design

Our medical oncology service is part of a military treatment facility that provides care for more than 240,000 beneficiaries. From January 2016 to June 2016, we led service-wide didactics in a quality improvement effort to introduce physicians in our clinic to the use of CARG to better identify patients who are considered “fit” for conventional therapy versus those who are “frail” and may experience an increased incidence of therapy-related complications. In July 2016, we asked medical oncologists to incorporate the tool as standard practice when considering giving chemotherapy to older patients with cancer. Physicians were instructed to paste results generated by the online calculator into their encounter notes and document any modifications to standard management based on the score.

Starting in May 2017, we retrospectively reviewed the electronic health records (EHR) of patients referred to our clinic. Our study included patients 75 years of age and older with initial history and physical examination documentation dated January 2016 to December 2016. All solid tumor and hematologic malignancy diagnoses that indicate the use of chemotherapy as a standard first-line therapy were included. We closely examined physician notes associated with each referral leading to any systemic antineoplastic therapy, spanning from initial history and physical examination to the start of therapy. We noted whether CARG was documented in connection with any physician encounter during that time span.

When CARG was documented, we assessed how physicians discussed it within their narratives of treatment recommendations. We included cases in which standard therapy was recommended without explicit regard to a documented CARG, cases resulting in recommendations against therapy based on clinical assessment of CARG, and cases in which treatment was modified at least partially based on CARG (i.e., dose reductions or regimen alterations). Lastly, we evaluated patients’ courses of care, including EHR data on hospitalizations and ED visits within six months after their initial encounters with medical oncology.

Statistical analysis and ethical approval

We reported utilization as a percent of chemotherapy recipients with a documented CARG. A chi-square test was performed to compare utilization during the six months before CARG adoption as a standard practice with utilization during the six months after adoption. Means and standard deviations were used as summary statistics for continuous variables, including the number of admissions, number of admission days, and number of ED visits. Kruskal-Wallis test p-values were calculated across continuous variables to compare patients with documented CARG who received standard therapy with patients whose CARG contributed to modifications in care. This study was approved by our institutional review board and deemed to constitute minimal risk.

## Results

**Figure 1:**
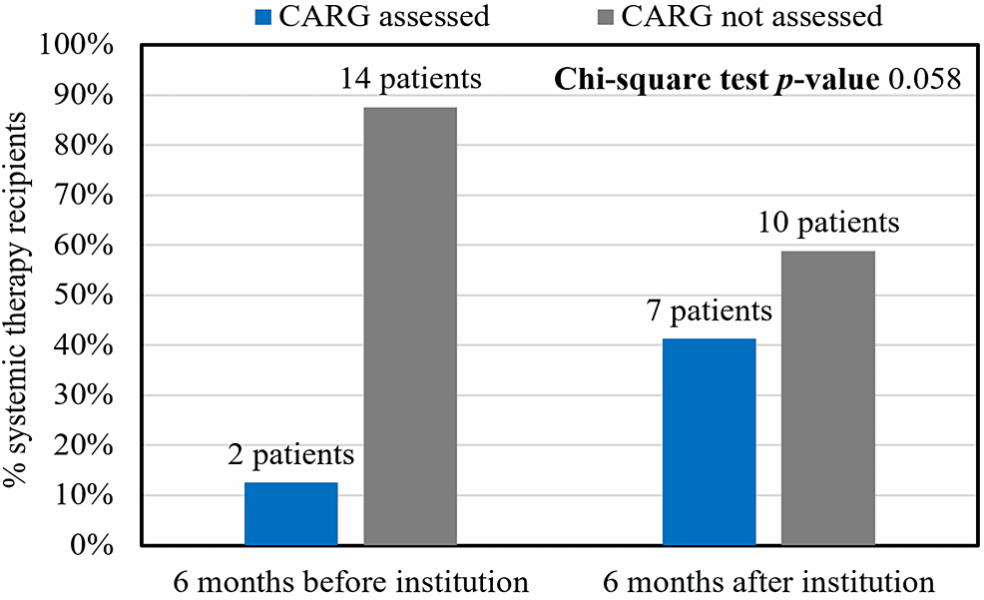
CARG utilization in systemic therapy recipients before and after its institution as a standard practice. CARG, Cancer and Aging Research Group toxicity prediction score

Thirty-eight patients who were newly referred to medical oncology met the inclusion criteria. Their mean age was 81.2 years, and 52.6% of them were male. The distribution of oncologic diagnoses included gastrointestinal (36.8%), lung (21.1%), hematologic (18.4%), breast (10.5%), genitourinary (2.6%), and other (10.5%) malignancies (Table 1). CARG occurred in 12.5% of systemic therapy recipients during the six-month period before its institution as a standard practice and 41.2% of systemic therapy recipients during the six-month period after its institution (Figure 1). There was no statistically significant difference in CARG utilization between the six-month period before its institution and the six-month period after its institution (p = 0.058), but there was a meaningful trend toward increased use. Physicians identified the difficulty of calculating CARG during time-constrained visits as the main barrier to its use.

**Table 1:** Patient characteristics (N = 38). x̅, mean; N, number of patients

Characteristic	Number of patients
Years of age (x̅ = 81.2)	
75-79	15 (39.5%)
80-84	14 (36.8%)
85-89	7 (18.4%)
90-94	1 (2.6%)
95-99	1 (2.6%)
Sex	
Female	18 (47.4%)
Male	20 (52.6%)
Type of cancer	
Gastrointestinal	14 (36.8%)
Lung	8 (21.1%)
Hematologic	7 (18.4%)
Breast	4 (10.5%)
Genitourinary	1 (2.6%)
Other	4 (10.5%)
CARG assessed	
Yes	14 (36.8%)
No	24 (63.2%)
Receipt of systemic therapy	
Yes	33 (86.8%)
No	5 (13.2%)

**Figure 2:**
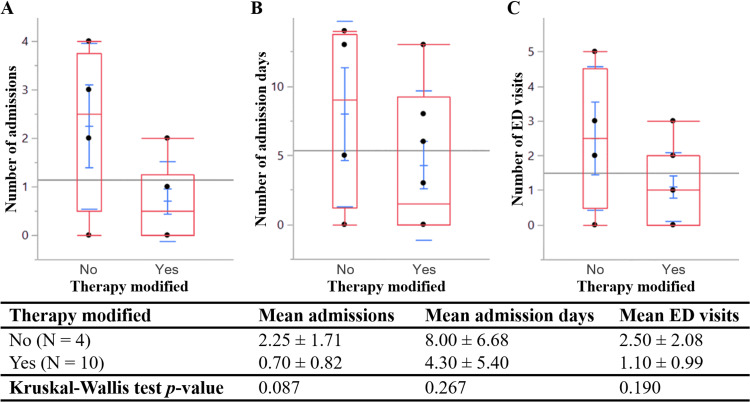
Box plots and summary statistics of (A) admissions, (B) admission days, and (C) ED visits by therapy modifications based on CARG. CARG, Cancer and Aging Research Group toxicity prediction score; ED, emergency department; N, number of patients

In 2016, CARG was employed in 14 patients, nine of whom received systemic therapy (Table 2) (Figure 1). Oncologic management was modified in 10 of 14 patients (71.4%) with documented CARG. Chemotherapy was initiated with dose reductions or other regimen alterations in five patients (35.7%) to minimize potential toxicities. The oncologist determined the need to forego chemotherapy, at least partially due to CARG, in another five patients (35.7%). Patients whose care was modified based on CARG (Table 2) had fewer mean admissions, days of admission, and ED visits (Figure 2) than those who received standard therapy. However, the study lacked power to establish statistical significance for admissions (p = 0.087), days of admission (p = 0.267), and ED visits (p = 0.19). One of nine (11.1%) systemic therapy recipients with documented CARG received single-agent immunotherapy, compared with none of the recipients without a documented CARG (Tables 2, 3). Two of nine (22.2%) systemic therapy recipients with a documented CARG enrolled in hospice within six months of initial evaluation, compared with three of 24 (12.5%) recipients without a documented CARG.

**Table 2:** Patients with documented CARG (N = 14). CARG, Cancer and Aging Research Group toxicity prediction score; DLBCL, diffuse large B-cell lymphoma; HCC, hepatocellular carcinoma; N, number of patients; NSCLC, non-small cell lung cancer

Age	Gender	Diagnosis	Total risk based on CARG	Systemic therapy	Treatment modification based on CARG	Hospice enrollment
86	Female	Anal carcinoma	Medium	Chemotherapy	Fluorouracil monotherapy, instead of multi-agent chemotherapy	No
85	Male	NSCLC	Medium	Chemotherapy	Did not offer concurrent chemoradiotherapy	Yes
98	Male	Bladder cancer	Medium	None	Systemic therapy not recommended	Yes
82	Male	HCC	Medium	Multikinase inhibitor	No modification	No
90	Male	DLBCL	High	None	Systemic therapy not recommended	Yes
83	Male	Colon cancer	Medium	Chemotherapy	Dose-reduced capecitabine monotherapy, instead of multi-agent chemotherapy	No
82	Female	Multiple myeloma	High	Immunomodulatory	Dose-reduced lenalidomide monotherapy	Yes
75	Female	NSCLC	Medium	Chemotherapy	No modification	No
77	Female	Rectal cancer	High	Chemotherapy	Omitted bevacizumab	No
78	Male	Gastric cancer	High	None	Systemic therapy not recommended	Yes
86	Female	NSCLC	Low	Immunotherapy	No modification	No
79	Male	DLBCL	Medium	Multi-agent	No modification	No
80	Female	Breast cancer	Medium	None	Systemic therapy not recommended	No
84	Male	Cutaneous melanoma	Medium	None	Systemic therapy not recommended	No

**Table 3:** Patients without documented CARG who received systemic therapy (N = 24). CARG, Cancer and Aging Research Group toxicity prediction score; CML, chronic myeloid leukemia; EGFR, epidermal growth factor receptor; DLBCL, diffuse large B-cell lymphoma; HCC, hepatocellular carcinoma; N, number of patients; NSCLC, non-small cell lung cancer; SCC, squamous cell carcinoma; SCLC, small cell lung cancer

Age	Gender	Diagnosis	Systemic therapy	Hospice enrollment
79	Female	Multiple myeloma	Immunomodulatory	No
77	Female	Breast cancer	Chemotherapy	No
77	Female	Rectal cancer	Chemotherapy	No
77	Female	Pancreatic cancer	Chemotherapy	No
81	Male	NSCLC	Chemotherapy	No
75	Male	HCC	Multikinase inhibitor	No
86	Male	Oropharyngeal SCC	EGFR inhibitor	No
80	Female	Breast cancer	Multi-agent	No
75	Female	Multiple myeloma	Multi-agent	No
80	Female	Cancer of unknown primary	Chemotherapy	Yes
85	Male	Esophageal cancer	Chemotherapy	No
80	Female	DLBCL	Multi-agent	No
86	Female	NSCLC	Chemotherapy	No
86	Male	NSCLC	Chemotherapy	No
76	Male	CML	Tyrosine kinase inhibitor	Yes
79	Female	Breast cancer	Chemotherapy	No
77	Female	SCLC	Chemotherapy	No
80	Male	NSCLC	Chemotherapy	No
81	Male	Laryngeal SCC	EGFR inhibitor	No
79	Male	Colon cancer	Chemotherapy	No
82	Male	HCC	Multikinase inhibitor	Yes
80	Male	Base of the tongue SCC	Chemotherapy	No
79	Female	Ileocecal cancer	Chemotherapy	No
84	Male	Pancreatic cancer	Chemotherapy	No

## Discussion

**Figure 3:**
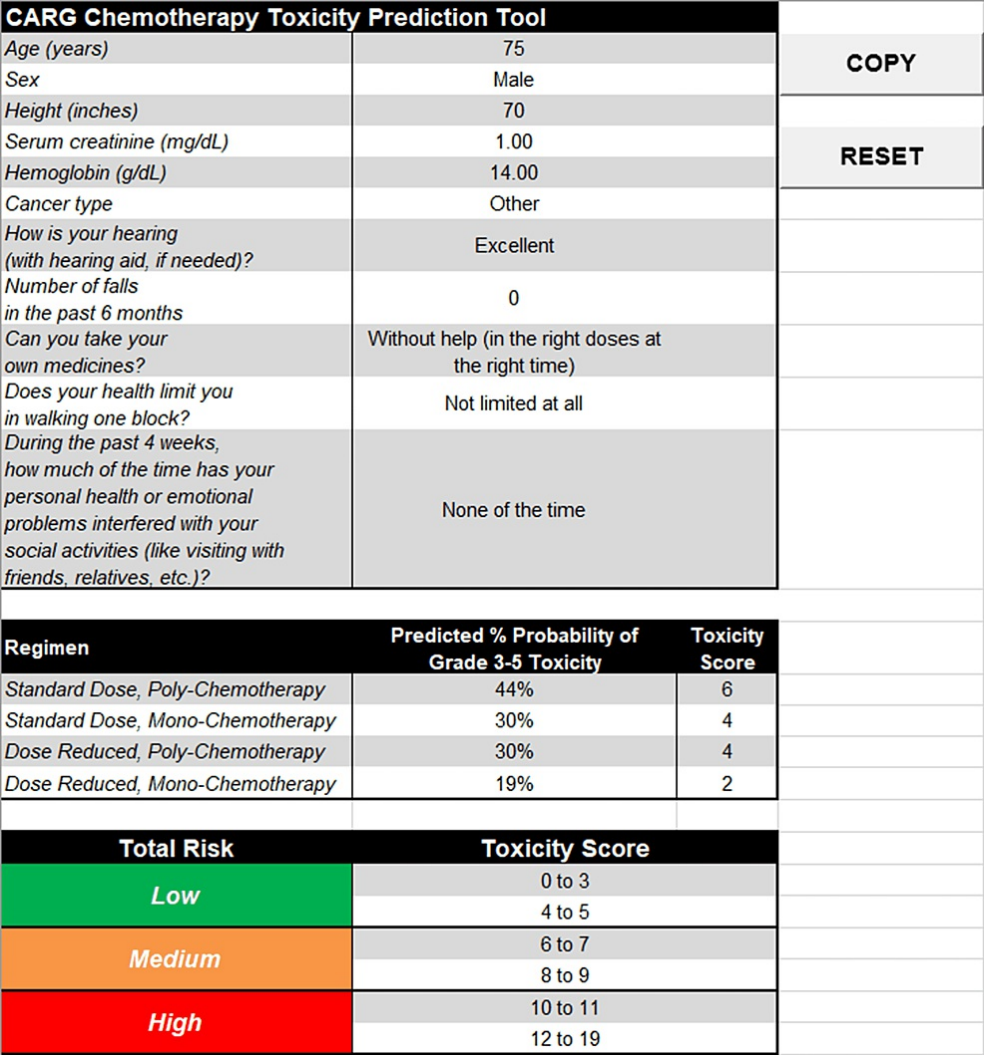
Spreadsheet-based CARG calculator tool. CARG, Cancer and Aging Research Group toxicity prediction score

Older adults with cancer constitute a diverse group of individuals with a wide range of comorbidities. They are particularly susceptible to a hospital-acquired disability, which could lead to increased rates of systemic therapy discontinuations and frequent interruptions in their cancer care. They are also particularly underrepresented in clinical trials, so the cumulative limitations on their treatment options likely result in reduced access to equitable care and underscore the importance of personalizing their management [3-5]. Consequently, it is important to employ validated tools such as CARG when planning appropriate treatment strategies. The utilization of CARG in our institution was limited after its implementation as a standard practice, and the score was documented in less than 50% of referrals leading to systemic therapy. However, its acceptance and use by medical oncologists increased over time. The exclusive reliance on physicians for CARG scoring in assessing older patients was an important barrier to its adoption. Implementing score calculations into patient screening would expand its application in cancer care. While training nonphysician team members may be resource-intensive, it is feasible, given the successful adoption of similar modalities such as depression severity screening. The online calculator is easily accessible, but scoring may be further simplified by including it in EHR systems or calculating CARG through automated spreadsheets such as the one we created with the assistance of the Cancer and Aging Research Group (Figure 3).

Immunotherapy has a growing role in the treatment of patients who are not candidates for systemic chemotherapy. One of the patients in our study who underwent CARG assessment received single-agent immunotherapy. CARG played no role in the selection of this therapy, but her case highlights the important point that CARG has not been validated in the context of single-agent immunotherapy. The evolving landscape of immuno-oncology requires the development of predictive tools capable of identifying older adults at risk for serious immunotherapy-related toxicity. Reassuringly, efforts are underway to examine the value of frailty assessments and screening tools such as the Geriatric 8 Score, which was predictive of hospital admissions and risk of death in a prospective cohort study by Gomes et al. [8]. Such tools could provide additional objective data points during difficult goals of care discussions. In the appropriate context, patients at objectively high risk for toxicity from both chemotherapy and immunotherapy could elect to pursue the best supportive care, rather than antineoplastic therapies.

Objectively risk-stratifying patients based on predicted toxicity from possible oncologic therapy can affect treatment recommendations, but the ultimate plan of care follows shared decision-making. Treatment may be declined for a multitude of reasons despite having a good prognosis and being at low risk for complications, including fears of decrements in quality of life. Beneficiaries in our health system are eligible for hospice services while undergoing cancer treatments. A higher percentage of therapy recipients enrolled in hospice within the six months after an initial evaluation when a CARG assessment was performed. Although this is difficult to interpret in our underpowered study, the assessment and its discussion with patients may facilitate the early pursuit of home-based palliation. Descriptive measures such as the average number of inpatient admissions, admission days, and ED visits may further inform patients of the impacts of therapy-related toxicity and encourage enrollment in hospice as a way to minimize their risks of hospitalization and pursue therapies that may otherwise seem unfeasible.

Although CARG assessments were not performed by all physicians, we found that their use in guiding management may decrease the risks of hospitalization in older patients with cancer. The power of this study was limited by restrictions in the inclusion age range and designated time span. We lacked a sufficient sample size to establish statistical significance, but modifying care based on CARG was associated with approximately half of the number of admissions, admission days, and ED visits when compared with standard therapy regardless of CARG. A 50% increase in sample size would establish statistical significance with a power of 80% if the current trend is preserved, so we deem our findings clinically meaningful. Variations in documentation practices also limit the accuracy of our findings, so standardized templates would be essential in future investigations.

## Conclusions

Older patients with cancer can be better served by a tailored treatment strategy that objectively addresses their substantial vulnerability to therapy-related toxicities. CARG is a validated measure that is suitable to serve as a standard frailty assessment tool, but its greatest value may rest in its predictive capacity to enable personalized planning. The trends that we observed helped us identify the number of hospitalizations as a pivotal metric that may be directly influenced by objectively guiding chemotherapy planning using CARG, rather than PS scales alone. Ultimately, an increased level of therapy personalization may open therapeutic options that were previously not accessible to older patients, so we will continue our work on a larger scale in an effort to enhance their access to high-quality equitable care.
